# Cholesterol-Lowering Drugs and Incident Open-Angle Glaucoma: A Population-Based Cohort Study

**DOI:** 10.1371/journal.pone.0029724

**Published:** 2012-01-04

**Authors:** Michael W. Marcus, Rogier P. H. M. Müskens, Wishal D. Ramdas, Roger C. W. Wolfs, Paulus T. V. M. De Jong, Johannes R. Vingerling, Albert Hofman, Bruno H. Stricker, Nomdo M. Jansonius

**Affiliations:** 1 Department of Ophthalmology, University Medical Center Groningen, University of Groningen, Groningen, The Netherlands; 2 Department of Epidemiology, Erasmus Medical Center, Rotterdam, The Netherlands; 3 Department of Ophthalmology, Erasmus Medical Center, Rotterdam, The Netherlands; 4 Department of Ophthalmogenetics, Netherlands Institute for Neuroscience, Amsterdam, The Netherlands; 5 Department of Ophthalmology, Academic Medical Center, Amsterdam, The Netherlands; 6 Department of Internal Medicine, Erasmus Medical Center, Rotterdam, The Netherlands; 7 Department of Medical Informatics, Erasmus Medical Center, Rotterdam, The Netherlands; Institute Biomedical Research August Pi Sunyer (IDIBAPS) - Hospital Clinic of Barcelona, Spain

## Abstract

**Background:**

Open-angle glaucoma (OAG) is a progressive neurodegenerative disease that may lead to blindness. An elevated intraocular pressure (IOP) is its major risk factor. OAG treatment is currently exclusively directed towards the lowering of the IOP. IOP lowering does not prevent disease progression in all patients and thus other treatment modalities are needed. Earlier studies reported cholesterol-lowering drugs to have neuroprotective properties. The aim of this study was to determine the associations between the use of cholesterol-lowering drugs and incident OAG.

**Methodology/Principal Findings:**

Participants in a prospective population-based cohort study underwent ophthalmic examinations, including IOP measurements and perimetry, at baseline and follow-up. The use of statins and non-statin cholesterol-lowering drugs was monitored continuously during the study. Associations between the use of cholesterol-lowering drugs and incident OAG were analyzed with Cox regression; associations between cholesterol-lowering drugs and IOP at follow-up were analyzed with multiple linear regression. During a mean follow-up of 9.8 years, 108 of 3939 eligible participants (2.7%) developed OAG. The hazard ratio for statin use was 0.54 (95% confidence interval 0.31–0.96; P = 0.034) and for non-statin cholesterol-lowering drugs 2.07 (0.81–5.33; P = 0.13). The effect of statins was more pronounced with prolonged use (hazard ratio 0.89 [0.41–1.94; P = 0.77] for use two years or less; 0.46 [0.23–0.94; P = 0.033] for use more than two years; P-value for trend 0.10). The analyzes were adjusted for age and gender, baseline IOP and IOP-lowering treatment, the family history of glaucoma, and myopia. There was no effect of statins on the IOP.

**Conclusions/Significance:**

Long-term use of statins appears to be associated with a reduced risk of OAG. The observed effect was independent of the IOP. These findings are in line with the idea that statins have neuroprotective properties and may open a way to a new OAG treatment modality.

## Introduction

Open-angle glaucoma (OAG) is a progressive neurodegenerative disease that leads to glaucomatous optic neuropathy and eventually, through glaucomatous visual field loss, to loss of sight. Together with age-related maculopathy it is the most common cause of irreversible blindness. An elevated intraocular pressure (IOP) is the major risk factor of OAG, and OAG treatment is currently exclusively directed towards the lowering of the IOP. However, OAG progression often continues despite an apparently sufficient reduction of the IOP. For that reason, the search for other OAG treatment modalities is a very active field of research.

Statins are selective inhibitors of 3-hydroxyl-3-methylglutaryl coenzyme A reductase (HMG-CoA) [1]. Currently, they are the most important lipid lowering medications for the treatment of hypercholesterolemia [2]–[4]. Previous studies have reported beneficial effects of statins on a variety of eye diseases, including age-related maculopathy, cataract and diabetic retinopathy [5]–[11]. Several observational studies addressed the effects of statins on OAG. Some reported a protective effect [12]–[14] whereas others did not [15], [16]. Studies including animal models as well as clinical trials have reported neuroprotective properties of statins [17]–[22]. Since OAG is characterized by the loss of neuronal cells, the use of statins, and possibly non-statin cholesterol-lowering drugs (NSCLDs) as well, might modify the risk of OAG through neuroprotection. With the current recommendations of lower primary prevention thresholds [23], [24], the use of statins and NSCLDs has increased markedly over the years [25]. For these reasons, it is expedient to clarify the associations between these drugs and OAG.

The aim of the present study was to determine the associations between the use of cholesterol-lowering drugs and incident OAG in a large prospective population-based cohort study.

## Methods

### Ethics statement

All measurements were conducted after the Medical Ethics Committee of the Erasmus University Rotterdam had approved the study protocol and all participants had given written informed consent in accordance with the declaration of Helsinki.

### Study population

The present study was performed as part of the Rotterdam Study, a prospective population-based cohort study investigating age-related disorders. The study population consisted of 7983 individuals aged 55 years and older living in the Ommoord district of Rotterdam, the Netherlands [26]. For this study, data from 3939 participants who did not have OAG (see below) at baseline and who completed at least one follow-up examination were used. The baseline examination took place from 1991 to 1993; follow-up examinations were performed from 1997 to 1999 and from 2002 to 2006.

### Ophthalmic assessment

Participants underwent similar eye examinations at baseline and at the two follow-up rounds [27]. These examinations included refraction, measurement of the best-corrected visual acuity, Goldmann applanation tonometry (Haag-Streit AG, Bern, Switzerland), fundoscopy, fundus photography of the posterior pole, simultaneous stereoscopic fundus photography of the optic disc, and visual field testing.

At each visit, three IOP measurements were taken on each eye and the median value of these three measurements was recorded [28]; the higher median of both eyes was used in the analysis. The visual field of each eye was screened using a 52-point supra-threshold test that covered the central visual field with a radius of 24° (Humphrey Field Analyzer [HFA]; Carl Zeiss, Oberkochen, Germany) [27], [29]. Visual field loss was defined as non-response to a light stimulus of 6 dB above a threshold-related estimate of the hill of vision in at least three contiguous test points, or four including the blind spot. In participants with reproducible abnormalities on supra-threshold testing, Goldmann perimetry (Haag-Streit AG, Bern, Switzerland; baseline and first follow-up) or full-threshold HFA 24-2 testing (second follow-up) was performed on both eyes. Visual field loss was considered to be glaucomatous visual field loss only if reproducible and after excluding all other possible causes [29], [30].

### Incident open-angle glaucoma

We defined incident OAG as no glaucomatous visual field loss in both eyes at baseline and glaucomatous visual field loss in at least one eye at follow-up [30]. All identified cases were examined by an experienced ophthalmologist (PTVMdJ and RCWW) who performed gonioscopy and a dilated ophthalmic exam. Cases with a history or signs of angle closure or secondary glaucoma were excluded.

### Medication data

Data on cholesterol-lowering drugs prescriptions for all participants were obtained from seven fully automated pharmacies using a centralized computer network in the study district, from January 1, 1991, onward. This included the product name, Anatomical Therapeutic Chemical (ATC) code, duration of use, and the date of first prescription. Cholesterol-lowering drugs were classified as statins (C10AA; simvastatin, pravastatin, fluvastatin, atorvastatin, cerivastatin, rosuvastatin) or NSCLDs (C10AB, C10AC, C10AD, C10A; fibrates, bile acid-binding resins or nicotinic acid and derivatives). The use of cholesterol-lowering drugs was recorded as the number of days with use during follow-up. Usage before baseline was not taken into account.

### Other covariates

Other covariates included age, gender, smoking, diabetes mellitus, cardiovascular diseases, the use of antihypertensive drugs, body mass index, total cholesterol, IOP, IOP-lowering treatment, and family history of glaucoma. All these covariates were measured at baseline. Smoking status was self reported and categorized as ever or never smoker. Data on diabetes mellitus and cardiovascular disorders such as angina pectoris, atrial fibrillation, myocardial infarction, heart failure, hypertension, and stroke were obtained from the participants through interviews, electrocardiogram readings, and non-fasting and fasting serum blood glucose levels. Diabetes was defined as the use of antidiabetic medication or by a non-fasting or post-load plasma glucose level above 200 mg/dl (11.1 mmol/l). Hypertension was defined as the use of antihypertensive medication for the indication of hypertension or as a systolic blood pressure of 140 mmHg or more, or a diastolic pressure of 90 mmHg or more. Body mass and height were measured at the research center. Total serum cholesterol was measured in non-fasting blood. IOP-lowering treatment was defined as the use of IOP-lowering medication or a history of glaucoma surgery or laser trabeculoplasty. The family history of glaucoma was determined by interviews and was considered positive if the participant reported a history of glaucoma in parents, siblings or offspring. Myopia was defined as a spherical equivalent refractive error of −4 D and more myopia [30]. Eyes with a cataract extraction before baseline were excluded from this analysis. In cases with one eye with incident OAG, the refraction of that eye was used. In participants without OAG or OAG in both eyes, the refraction of a random eye was used.

### Statistical analysis

Differences in baseline characteristics between participants with and without incident OAG and differences in baseline characteristics between cholesterol-lowering drug users and non-users were evaluated using chi-square tests for categorical variables and t-tests for normally distributed continuous variables. To determine the associations between the use of cholesterol-lowering drugs and incident OAG, the use of statins or NSCLDs was initially defined as any use during follow-up and the associations were initially analyzed with chi-square tests. Subsequently, a Cox proportional hazards model was used to calculate hazard ratios (HR) and corresponding 95% confidence intervals (CI) for the associations between the use of statins or NSCLDs and incident OAG. Follow-up duration was used as the time axis in the model. For participants without incident OAG, the follow-up duration was counted from the baseline visit to the last visit with reliable perimetry. For incident OAG cases, the follow-up ended at the first visit in which glaucomatous visual field loss was detected. The cholesterol-lowering drugs, age and gender, and other covariates with P<0.20 in the univariate comparisons were included in the multivariate analysis. Subsequently, the cholesterol-lowering drugs, age and gender, and other covariates with P<0.05 in the initial multivariate model were included in the final model. The use of cholesterol-lowering drugs was entered in the model as any use during follow-up. To allow for the evaluation of a possible dose-response relationship, we also performed analysis after making three nominal categories based on the duration of medication use, being no use, cumulative use during two years or less, and cumulative use during more than two years (see Discussion section). The dose-response relationship was evaluated with a trend test. To explore the influence of cholesterol-lowering drugs on the IOP, we conducted a multiple linear regression analysis with IOP at follow-up as the dependent variable. This analysis was adjusted for IOP-lowering treatment at follow-up and for the same covariates as the final Cox model except for baseline IOP and IOP-lowering treatment at baseline. All analyzes were performed using SAS 9.2 (SAS Institute Inc., Cary, NC). A *P*-value of 0.05 or less was considered statistically significant.

## Results

During a mean follow-up of 9.8 years, 108 of 3939 eligible participants (2.7%) developed OAG. Table 1A depicts the baseline characteristics of the study population for participants with and without incident OAG. Participants with incident OAG were older, more often male, more often had a positive family history of glaucoma, and more often had myopia. They also had a higher IOP and more frequently received IOP-lowering treatment. There was no difference between the groups regarding total serum cholesterol levels. Table 1B shows the baseline characteristics for cholesterol-lowering drug users and non-users. Participants using cholesterol-lowering drugs were younger, smoked less frequently and more often had diabetes mellitus, a myocardial infarction or hypertension. They also used more often antihypertensive drugs and had a higher total serum cholesterol level and a slightly higher IOP.

**Table 1:** Baseline characteristics of participants with and without incident open-angle glaucoma (A) and of cholesterol-lowering drug users (either statins or NSCLDs, or both) and non-users (B), with univariable comparisons (mean values with standard deviation between brackets unless stated otherwise).

A	Incident open-angle glaucoma(N = 108)	No incident open-angle glaucoma(N = 3831)	P-value
Age (year)	68.4(7.1)	65.7(6.8)	<0.001
Gender (%female)	49.1	58.7	0.046
Smoking (%)	33.3	33.4	0.98
Diabetes mellitus (%)	8.4	6.9	0.54
Angina pectoris (%)	1.9	3.1	0.46
Atrial fibrillation (%)	2.8	2.1	0.63
Myocardial infarction (%)	13.2	9.7	0.23
Heart failure (%)	0.9	1.2	0.81
Hypertension (%)	52.9	47.1	0.49
Blood pressure lowering drugs (%)	28.0	26.0	0.63
Stroke (%)	2.8	1.2	0.16
Body mass index (kg/m^2^)	25.8(2.9)	26.3(3.5)	0.12
Total cholesterol (mmol/l)	6.5(1.1)	6.7(1.2)	0.17
IOP (mmHg)	17.3(4.7)	15.0(3.1)	<0.001
IOP-lowering treatment (%)	15.7	2.3	<0.001
Family history of glaucoma (%)	16.7	8.1	0.002
Myopia	9.5	4.9	0.033

B	Cholesterol-lowering drug users(N = 848)	Non-users(N = 3091)	
Age	64.3(5.5)	66.1(7.1)	<0.001
Gender (%female)	56.6	58.9	0.23
Smoking (%)	27.5	35.0	<0.001
Diabetes mellitus (%)	10.2	6.1	<0.001
Angina pectoris (%)	3.8	2.8	0.15
Atrial fibrillation (%)	2.4	2.1	0.57
Myocardial infarction (%)	15.3	8.2	<0.001
Heart failure (%)	1.2	1.2	0.97
Hypertension (%)	58.7	47.1	<0.001
Blood pressure lowering drugs (%)	38.3	22.6	<0.001
Stroke (%)	1.4	1.2	0.65
Body mass index (kg/m^2^)	26.5(3.5)	26.2(3.5)	0.055
Total cholesterol (mmol/l)	7.4(1.3)	6.5(1.1)	<0.001
IOP (mmHg)	15.3(3.2)	15.0(3.2)	0.044
IOP-lowering treatment (%)	2.0	2.9	0.18
Family history of glaucoma (%)	9.7	8.0	0.13
Myopia	6.3	4.7	0.063

NSCLDs = non-statin cholesterol-lowering drugs; IOP = intraocular pressure.

Table 2 presents the results of the univariable analyses for the use of statins and NSCLDs at any time during follow-up. These univariable comparisons revealed no significant differences between participants with and without incident OAG. Amongst the 811 participants using statins at any time during follow-up, the median duration of use was 1424 days, with a range from 8 to 4114 days; amongst the 113 participants using NSCLDs, the median duration of use was 298 days, with a range from 7 to 3544 days.

**Table 2:** Univariable analyses of the use of cholesterol-lowering medication at any time during follow-up and the development of open-angle glaucoma.

	Incident open-angle glaucoma (N = 108)	No incident open-angle glaucoma (N = 3831)	P-value
Statins (n[%])	16(14.8)	795(20.8)	0.13
NSCLDs (n[%])	5(4.6)	108(2.8)	0.27

NSCLDs = non-statin cholesterol-lowering drugs.

Table 3 presents the final multivariate model, adjusting for age and gender, baseline IOP and IOP-lowering treatment, the family history of glaucoma, and myopia. Participants using statins had a significant risk reduction (HR 0.54; 95% CI 0.31 to 0.96; P = 0.034). The use of NSCLDs was not significantly associated with incident OAG (HR 2.07; 95% CI 0.81 to 5.33; P = 0.13). There was a trend towards a reduced risk of incident OAG with prolonged statin use. The HR was 0.89 (95% CI 0.41 to 1.94, P = 0.77) for use during two years or less and 0.46 (95% CI 0.23 to 0.94, P = 0.033) for use during more than two years. The overall P-value for trend was 0.10.

**Table 3:** Final multivariate model of the risk of developing open-angle glaucoma for cholesterol-lowering medication.

	Hazard ratio	95% confidence interval	P-value
Statins	0.54	0.31–0.96	0.034
NSCLDs	2.07	0.81–5.33	0.13
Age (per year)	1.07	1.04–1.10	<0.001
Gender (female)	0.56	0.38–0.83	0.004
IOP (per mmHg)	1.12	1.08–1.18	<0.001
IOP treatment	3.39	1.82–6.32	<0.001
Family history of glaucoma	1.85	1.08–3.15	0.024
Myopia	2.30	1.19–4.43	0.013

NSCLDs = non-statin cholesterol-lowering drugs; IOP = intraocular pressure.

The protective effect of statins could be either caused by an IOP-lowering effect of statins or by a direct protective effect of statins on the neural tissue. To differentiate between these two possibilities, we conducted a multiple linear regression analysis with IOP at follow-up as the dependent variable. Table 4 shows the results. As can be seen in this table, there was no significant IOP-lowering effect of statins.

**Table 4:** Multiple linear regression analysis with intraocular pressure at follow-up as the dependent variable.

	beta	95% confidence interval	P-value
Statins	−0.006	−0.262–0.249	0.96
Age (year)	−0.011	−0.026–0.005	0.18
Gender (female)	−0.269	−0.479–−0.060	0.012
IOP-lowering treatment at follow-up	1.761	1.340–2.181	<0.001
Family history of glaucoma	0.378	0.001–0.755	0.050
Myopia	0.597	0.124–1.069	0.013

IOP = intraocular pressure.

## Discussion

In this large prospective population-based study, the use of statins was associated with a reduced risk of OAG. This effect was independent of the IOP. The risk reduction tended to increase with the duration of cumulative use, which supports the observed association, but this trend did not reach statistical significance. The use of NSCLDs was not associated with the development of OAG.

The association between the use of statins and OAG we found is consistent with the results of McGwin et al. (odds ratio 0.60; 95% CI 0.39 to 0.92) [12]. They performed a nested case-control study in a clinical administrative database. In contrast, Owen et al. found no evidence for a protective effect of statins (odds ratio 0.97; 95% CI 0.88 to 1.06) [15]. They employed a case-control study design in a primary care database. Similarly, Iskedjidan et al. did not find a significant association between the use of statins and OAG [16]. They performed a retrospective population-based evaluation in an administrative prescription claims database. The designs of the latter two studies might have complicated the classification of OAG, and the resulting misclassification might have biased the effect estimate. The trend of the effect seen in our study is consistent with previously published studies. McGwin et al. reported a significant reduction in the risk of OAG in patients using statins for more than 23 months [12]. De Castro et al. reported that the use of statins was associated with a slower progression of glaucomatous optic nerve atrophy [14]. In their clinical retrospective cohort study, patients using statins for two years showed less optic nerve head changes than patients not using statins. Nagaoka, et al studied the effect of statins on the retinal circulation and the IOP [31]. They found an IOP decrease after the administration of statins. At first sight, this seems to corroborate with our findings. However, our data suggested the protective effect of statins to be IOP independent. A possible explanation for this discrepancy might be that they studied the effects of statins up to one week after the initial administration whereas we found the most pronounced effect in those OAG cases that used statins for more than two years. Leung et al. reported that the use of simvastatin was associated with visual field stabilization in patients with normal tension glaucoma (relative risk 0.36; 95% CI 0.14 to 0.91; P = 0.030) [13]. In their prospective cohort study, 256 patients with normal tension glaucoma of whom thirty-one were taking simvastatin and 225 were not taking simvastatin were followed-up for 36 months.

The use of NSCLDs was not associated with incident OAG in our study. This result contradicts the result of the study by McGwin et al. who found a protective effect among those who used NSCLDs (odds ratio 0.59; 95% CI 0.37 to 0.97) [12]. This discrepancy might be attributed to the low number of users of NSCLDS in our study, as depicted by the wide CIs.

Strengths of our study include its prospective design, the large number of participants, the long follow-up period and the population-based setting, which minimizes selection bias. An inextricable limitation of the population-based design is the limited number of OAG cases – due to the low prevalence of OAG - and the limited number of participants using NSCLDs. Information bias was prevented by prospectively collected and completely automated pharmacy records of all prescriptions. Although this approach guarantees accurate prescription data, it cannot be guaranteed that all participants actually took their medication. Such exposure misclassification is usually similar in cases and controls and leads to conservative risk estimates. Hence, it may have contributed to the lack of effect of NSCLDs, but not to the protective effect of statins.

Several other factors have been reported to be a risk factor for the incidence of OAG, including myopia [32], pseudoexfoliation [33], central corneal thickness [34], and age [30]. Of these factors, only pseudoexfoliation and age may be associated with statin use and may thus be confounding factors in our analysis [35]–[37]. Pseudoexfoliation is relatively rare in the Netherlands and in our study population (which might or might not be due to underreporting) – hampering a meaningful adjustment for pseudoexfoliation in our analysis. However, the absence of adjustment should have resulted in an increased risk whereas we found a protective effect. Age is associated with statin use but we adjusted our models for that. Age as a linear covariable – as we did – might result in under-adjustment, but in that case an increased risk should have been the result, not a protective effect. We included only participants aged 55 years and older. This is not a limitation for this specific study question, as both statin use at younger age is relatively rare and the prevalence of OAG below 55 years of age is very low (0.1–0.2%; to be compared to 1–2% above 55 years of age [27]. Finally, myopia appeared to occur – presumably by chance – slightly more frequent amongst cholesterol-lowering drug users compared to non-users (P = 0.063; Table 1B) and was included in the final model.

A possible limitation of this study is potential misclassification of exposure. However, such misclassification will be random because the outcome is – inextricably - gathered irrespective of exposure status. To appreciate this approach, it is important to realize that glaucoma development often takes more than a decade and cannot be detected in the earliest stages. Some factors slow down or accelerate the disease development, and thus make it less likely or more likely that the disease can be detected at a certain point in time (being our follow-up examination). Cumulative exposure stratified into biologically plausible nominal categories as we used in our analyses is the best proxy for studying the overall influence of the use of medication on the rate of glaucoma development during follow-up. Details of this technique were published earlier [38]. Because the exposure misclassification is random, it will tend to bias the results towards the null hypothesis. This might mean that the significant protection we found is an underestimation of the true effect.

Our finding of a protective effect of statins may offer potential therapeutic possibilities for OAG or its prevention. We showed the effect to be independent of the IOP. Hence, the protective effect of statins could be caused by lowering serum cholesterol or by (other) neuroprotective properties of statins on neuronal cells, as mentioned in the Introduction section [17]–[22]. Our incident OAG cases did not have an elevated serum cholesterol level at baseline (Table 1), but that observation does not exclude a beneficial effect of a further lowering of this level - cardiovascular trials have shown beneficial effects of further lowering cholesterol levels even if initially already within normal limits [39], [40]. Studies with serum cholesterol level monitoring during follow-up should enable the uncovering of more details of the mechanism underlying the protective effect of statins. Given the current level of evidence and the fact that statins are widely available and thoroughly investigated drugs, a neuroprotective OAG treatment could become reality and a randomised clinical trial seems to be a viable next step.

In conclusion, we confirmed that statins appear to have a protective effect on OAG. Due to our study design, we were able to add that this protective effect is IOP independent. Hence, statins should be further explored as a new class of medications for the treatment of OAG, especially for those patients in whom disease progression continues despite an apparently sufficient IOP reduction.
